# Gallie's Treatment of High Dorsal Potts' Disease

**Published:** 1910-01-08

**Authors:** 


### Surgery.

### GALLIE'S TREATMENT OF HIGH DORSAL POTTS' DISEASE.

The treatment of High Dorsal Potts' disease is always a matter of considerable difficulty. The reason is that the distance between the kyphosis and the top of the thorax is so short that any plan of treatment that depends upon pressure backwards on the upper dorsal vertebræ to support the weight of the head and shoulders necessarily fails because of the shortness of the leverage provided.

A plan of treatment has been devised as a means of getting over the mechanical difficulty presented. This involves the lengthening of the short upper lever by adding to it the cervical spine. Inasmuch as it is impossible to apply pressure on the front of the neck, owing to the presence of the larynx and vessels, the splint must be extended up to the head, and preferably to the forehead and occiput. It consists of a plaster jacket extending from the pelvis to the head, leaving free the arms and exposing the face, ears, and part of the chest. It conforms to the principles laid down for the treatment of tuberculosis of bone, in that it provides fixation to the parts, prevents increase of the deformity, and, when applied with moderate hyper-extension of the spine above and below the kyphosis, causes the deformity to diminish. The plan has been given a thorough trial extending over a period of two years, and so far the results have been most gratifying. All the cases have shown marked improvement, the acute symptoms disappearing rapidly after the application of the first jacket. In no case has the deformity increased, and in practically all there has been a diminution of the kyphosis.

The general plan of management of these cases includes both recumbent and ambulatory treatment. Although the jacket gives excellent support to the spine, nevertheless the patient is kept in bed six months, or more, as an extra precaution. After that, if there is reasonable improvement, he is allowed up. The length of time he will need to wear the jackets is a problem. A natural cure usually takes five or six years to establish itself, and the ordinary methods of treatment do not materially shorten this period. Dr. Gallie hopes to reduce this time substantially, and at the same time leave the patient with a straight back, but he will not be able to report definitely on this point until three or four years more have elapsed.

The method of application of the jacket was arrived at after considerable experiment. Most of the difficulties were overcome by the apparatus of an upright fork, the arms of which are drilled out to form a socket. On the top of the fork rests a plate through which pass two pins, each of which fits into the socket in one of the arms of the fork. Thus the plate is secured against lateral movement, and may yet be lifted free at will. To this plate are sewn two strips of Mexican felting, and the felt and plate are moulded to fit about the kyphosis and remove all pressure from the spines. The patient's head rests on a forked support, which is made to represent the position of one's arm and hand in supporting the head when the patient is in a horizontal position. The arms of the fork are long and thin, and are curved to conform roughly with the shape of the back of the head. The head can be raised or lowered, as can also the kyphosis, by thumbscrews on the uprights. The pelvis and legs rest on a padded box. The head-rest, spinal support and box are all placed on a slide, so that the apparatus is adjustable to any sized patient and to disease in any region.

The application of the stockinette requires special mention. The method adopted is to draw the stockinette up over the body and head and then cut it down the sides as far as the axillæ. Thus the stockinette is intact on the body, and consists of an anterior and posterior flap on the head and neck. These flaps are sewn together on the tops of the shoulders from the outer border of the material to the side of the neck. The seam then turns upwards and the two flaps are sewn together close to the side of the face and head. When the stitching is complete the material external to the seam is cut away and the head, neck, and shoulders are left exactly fitted. The patient is now turned on his face, and a strip of silence cloth about four inches wide is stitched to the stockinette, extending from the top of the head to the pelvis, the object being to protect the spines. The hips are also protected in a similar manner.

This completes the preliminary preparations, and the patient is placed on the jacket apparatus. The head and spinal supports are raised or lowered until the proper recession of the kyphosis by hyper-extension of the spine above and below has been obtained. An assistant holds the arms out from the sides and draws down the shoulders, and the operator proceeds with the plaster. Two or three four-inch bandages are first applied to the body, incorporating the padded kyphosis plate. Several narrow bandages are next applied to the head, fastening it securely to its support. The head and body parts of the jacket are then joined together by making thick straps of plaster, six or eight inches long, and laying them one on each side of the face and extending down on to the chest. A similar strap is applied to the back of the neck. The shoulders are covered over in the same way, and all the straps are fastened into place by circular bandages, which are worked into the uneven surfaces about the shoulders and neck. The plaster in this region is made very thick, about half an inch usually, as it is proposed to cut out large areas later for the sake of ventilation. The patient remains on the apparatus until the plaster has set, and is then freed by lifting him off the forked spinal support and slipping the head-support out from under his head. When the back of the jacket is examined, the two pins will be sticking out uncovered. These are cut off with a pair of ordinary wire cutters, and the area covered over with some plaster cream.

The cutting out is now proceeded with. A sharp knife is required, and considerable care must be exercised to avoid cutting the patient about the head. The face is completely freed in front, and care is taken to leave ample room for movement of the lower jaw. The scalp is uncovered and the ears exposed. If the plaster has been made sufficiently thick, the amount cut out can be increased until the head is simply enclosed in a series of plaster bars. The arms are made quite free by cutting out the plaster for two or three inches in the axillæ. Finally, a large opening is made over the chest, with a view to allowing free movement of the thorax. When the plaster has thoroughly dried another length of stockinette is drawn over the jacket and sewn to the inside layer at all the cut edges, so as to make a perfectly fitting cover. If care and skill have been employed, the final result will be a comfortable and efficient jacket, which is no more apt to attract public curiosity than any of the other apparatus used.

The length of time the jacket ought to last depends entirely on the patient. Some may be left on six months, while others must be renewed for sanitary reasons in half that time. In dealing with Potts' disease, however, one must be prepared to sacrifice cleanliness somewhat, because of the injury resulting from frequent manipulations. The plan of threading coarse bandages under the inside stockinette and see-sawing them daily over the skin will assist materially in keeping the skin healthy.

